# Fexinidazole: A Potential New Drug Candidate for Chagas Disease

**DOI:** 10.1371/journal.pntd.0001870

**Published:** 2012-11-01

**Authors:** Maria Terezinha Bahia, Isabel Mayer de Andrade, Tassiane Assíria Fontes Martins, Álvaro Fernando da Silva do Nascimento, Lívia de Figueiredo Diniz, Ivo Santana Caldas, André Talvani, Bernadette Bourdin Trunz, Els Torreele, Isabela Ribeiro

**Affiliations:** 1 Laboratório de Doença de Chagas, Departamento de Ciências Biológicas & Núcleo de Pesquisas em Ciências Biológicas, Universidade Federal de Ouro Preto, Campus Universitário, Morro do Cruzeiro, Ouro Preto, Brazil; 2 Drugs for Neglected Disease *initiative* (DNDi), Geneva, Switzerland; Northeastern University, United States of America

## Abstract

**Background:**

New safe and effective treatments for Chagas disease (CD) are urgently needed. Current chemotherapy options for CD have significant limitations, including failure to uniformly achieve parasitological cure or prevent the chronic phase of CD, and safety and tolerability concerns. Fexinidazole, a 2-subsituted 5-nitroimidazole drug candidate rediscovered following extensive compound mining by the Drugs for Neglected Diseases *initiative* and currently in Phase I clinical study for the treatment of human African trypanosomiasis, was evaluated in experimental models of acute and chronic CD caused by different strains of *Trypanosoma cruzi*.

**Methods and Findings:**

We investigated the *in vivo* activity of fexinidazole against *T. cruzi*, using mice as hosts. The *T. cruzi* strains used in the study were previously characterized in murine models as susceptible (CL strain), partially resistant (Y strain), and resistant (Colombian and VL-10 strains) to the drugs currently in clinical use, benznidazole and nifurtimox. Our results demonstrated that fexinidazole was effective in suppressing parasitemia and preventing death in infected animals for all strains tested. In addition, assessment of definitive parasite clearance (cure) through parasitological, PCR, and serological methods showed cure rates of 80.0% against CL and Y strains, 88.9% against VL-10 strain, and 77.8% against Colombian strain among animals treated during acute phase, and 70% (VL-10 strain) in those treated in chronic phase. Benznidazole had a similar effect against susceptible and partially resistant *T. cruzi* strains. Fexinidazole treatment was also shown to reduce myocarditis in all animals infected with VL-10 or Colombian resistant *T. cruzi* strains, although parasite eradication was not achieved in all treated animals at the tested doses.

**Conclusions:**

Fexinidazole is an effective oral treatment of acute and chronic experimental CD caused by benznidazole-susceptible, partially resistant, and resistant *T. cruzi*. These findings illustrate the potential of fexinidazole as a drug candidate for the treatment of human CD.

## Author Summary

This study describes the in vivo activity of fexinidazole against *Trypanosoma cruzi*, the protozoan parasite causing Chagas disease, using mice infected with parasite strains with varying susceptibility to benznidazole, the standard treatment for Chagas. Fexinidazole and benznidazole were shown to have similar activity against benznidazolesusceptible and partially resistant *T. cruzi* strains (CL and Y), but fexinidazole had potent activity against benznidazole-resistant strains (VL-10 and Colombian). Fexinidazole treatment resulted in parasitological cure during acute disease phase in 88.9% of mice infected with the VL-10 strain and 78% with Colombian strain; benznidazole treatment did not result in cure in animals infected with these strains. Fexinidazole treatment was also shown to reduce myocarditis in all VL-10- and Colombian-infected animals, although parasite eradication was not achieved in all treated animals. These data demonstrate that it is possible to achieved better cure rates with fexinidazole in these experimental infection models than what is achieved with the standard benznidazole at the doses tested in this animal study benznidazole treatment regimen.

## Introduction

One century after its discovery, American trypanosomiasis, or Chagas disease, remains a serious health problem in Latin America, where it affects 8–10 million people with 100 million at risk of acquiring the disease [1]. Chemotherapy, together with vector and transfusion control, is one of the most important elements in the control of Chagas disease, since no vaccine is yet available to prevent infection. Treatment is dependent solely on two drugs, benznidazole and nifurtimox, which have a number of drawbacks including toxicity, drug resistance, and insufficient effectiveness against chronic disease. Nevertheless, as of today, these drugs are the only available therapeutic options in endemic and non-endemic areas.

New potential treatment options include inhibitors of the sterol biosynthesis pathway, in particular C14-α-demethylase inhibitors such as posaconazole and ravuconazole, which represent promising new drugs candidates [2], [3]. Despite the superior *in vitro* potency and *in vivo* efficacy of these novel azole derivatives against *T. cruzi*, and the absence of cross-resistance with currently available drugs, response to drug treatment varies among the different strains of the parasite [3]–[6]. Also, key disadvantages of currently marketed novel azole derivatives, such as posaconazole, are the complexity and cost of manufacturing these compounds, which will need to be addressed to facilitate access to resource-limited populations that constitute the vast majority of patients with Chagas disease [7].

Therefore, the continued search for new trypanocidal treatments that could be effective for Chagas disease remains a priority for Chagas disease control. Nitroimidazoles are a well-known class of pharmacologically active compounds, among which several have shown good activity against trypanosomes [8]. While concerns over mutagenicity have mitigated their potential as drug candidates, several members of this family including metronidazole [9] are widely used as antibiotics, indicating that it is possible to select compounds with acceptable activity/toxicity profile in this class. Today, two non-mutagenic novel nitroimidazole-oxazine compounds (PA-824 and OPC-67683) are in clinical development for tuberculosis [10], while the 2-substituded 5-nitroimidazole fexinidazole is in clinical development for human African trypanosomiasis (also known as sleeping sickness) [11].

Fexinidazole (previously known as Hoe 239) had been in preclinical development as a broad-spectrum antiprotozoal drug by Hoechst in the 1970s–1980s, but its clinical development was not pursued at the time. The molecule was “rediscovered” and selected for development by the Drugs for Neglected Diseases *initiative* (DNDi) as a new drug candidate for sleeping sickness [11], following a systematic review and profiling of more than 700 nitroheterocyclic compounds (mostly nitroimidazoles) from diverse sources, which included assessments of antiparasitic activity and mutagenic potential. Fexinidazole underwent extensive regulatory toxicology studies, including safety pharmacology (respiratory, cardiovascular, and general behavior) and 4 weeks of repeated-dose toxicokinetics studies in rat and dogs. Overall, fexinidazole was found to be well tolerated, with no specific toxicity or other concerns [11]. During 2010–2011, DNDi carried out two Phase I clinical trials assessing the safety and pharmacokinetics of fexinidazole in human volunteers given in single and multiple doses. A phase II/III clinical safety and efficacy study in sleeping sickness patients is slated to begin in mid-2012.

Fexinidazole has previously been described as effective and superior to benznidazole or nifurtimox in one acute murine infection model with the *T. cruzi* Brazil 32 strain [12], but the methodologies used to establish cure are no longer considered the most accurate. Here were evaluated the *in vivo* activity of fexinidazole in mice infected with a panel of *T. cruzi* strains with differing levels of benznidazole susceptibility and looking at both acute and chronic infection, and using state of the art methods to establish cure.

## Materials and Methods

### Parasite Strains

The *Trypanosoma (Schizotrypanum) cruzi* strains Y (DTU II), CL (DTU VI), VL-10 (DTU II), and Colombian (DTU I) [13] were used in this study. Y strain, partially resistant to benznidazole, was used as the standard strain because it induces high parasitemia and 100% mortality, which is generally observed at days 10 to 19 post-infection. CL strain is highly sensitive to benznidazole, and VL-10 and Colombian strains are highly resistant to benznidazole.

### Study Drugs

**Figure 1:**
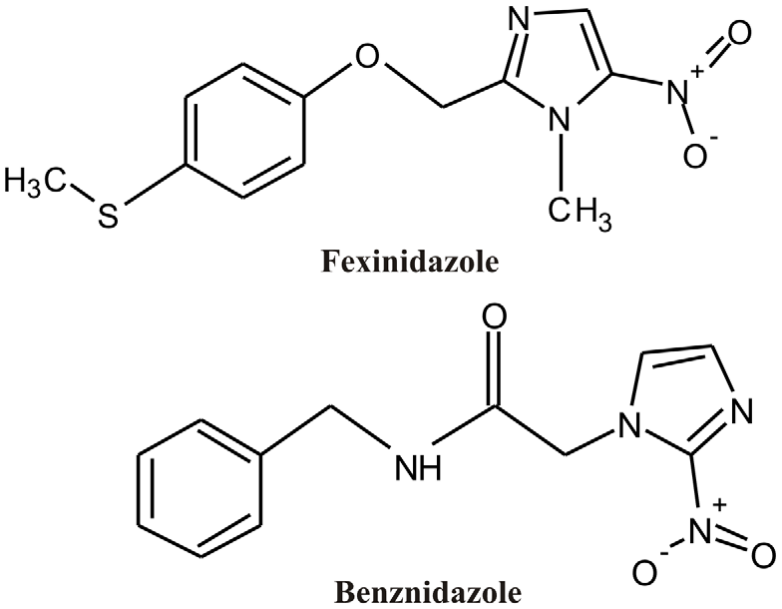
Chemical structure of fexinidazole and benznidazole.

Fexinidazole (1H-imidazole, 1-methyl-2-((4-(methylthio)phenoxy)methyl)-5-nitroimidazole) (Figure 1) was administrated orally in suspension containing methyl cellulose 0.5% w/v, with 5% v/v of polysorbate 80 (Tween 80). Benznidazole (2-nitroimidazole-(N-benzil-2-nitzo-1-imidazoleacetamide; Rochagan, Roche) (Figure 1) was used as the reference treatment in this study and was administered orally in a water suspension with 4% methyl cellulose. Cyclophosphamide (*N*,*N*-bis(2-chloroethyl)-1,3,2-oxazaphosphinan-2-amine 2-oxide; Genuxal, Asta Medica Oncologica) was diluted in ultrapure water and administered intraperitoneally. The treatment consisted of three cycles of 50 mg of cyclophosphamide/kg of body weight for four consecutive days with intervals of three days between each cycle.

### Animal Model

Female Swiss mice from the Animal Facility at Ouro Preto Federal University (UFOP), Minas Gerais State, Brazil were used in this study. Animals were fed with commercial food and water was available *ad libitum*. Swiss mice (18–20 g) were inoculated intraperitoneally with 5.0×10^3^ bloodstream trypomastigotes of the Y, CL, VL-10, and Colombian *T. cruzi* strains.

### Dose Response

Mice infected with *T. cruzi* Y strain (6 animals/group) were treated with doses of 100, 200, and 300 mg/kg of body weight (mpk) of fexinidazole per day. The drugs were administered orally on day 4 post-infection for 7 consecutive days. Drug efficacy was assessed based on three parameters: parasite clearance, time of absence of parasitemia, and mortality. All parameters were compared to those observed in benznidazole treatment using a standardized therapeutic scheme of 100 mpk per day [14].

### Determination of Efficacy by Parasitological Cure

The first set of experiments was designed to determine the efficacy of fexinidazole to induce parasitological cure in mice infected with Y strain (partially resistant to benznidazole). Groups of 10 mice infected with Y strain were treated with 4 different doses of fexinidazole (50, 100, 200, and 300 mpk of drug per day). The drug was administered at the time of parasitemia detection, which occurred at day 4 post-inoculation for 20 consecutive days. Parasitological cure was determined as described below, and the results were compared to those achieved with treatment with benznidazole [14]. A group of 10 animals infected with the parasite but receiving no treatment was used as control.

The second set of experiment was designed to determine the efficacy of fexinidazole to induce parasitological cure in mice infected with strains of different level of resistance to benznidazole: CL, VL-10, and Colombian. Because of the high benznidazole resistance of VL-10 and Colombian strains, the dose of fexinidazole used was the dose inducing the highest level of parasitological cure in animals infected with Y strain. The drug was administered at the time of parasitemia detection which occurred at day's 7–8 post-inoculation for 20 consecutive days. Parasitological cure was determinate as described below, and results were compared to those treated with benznidazole and with untreated control groups, infected or uninfected with parasites.

Additional experiments were performed in a murine model of chronic phase of the disease to confirm the therapeutic efficacy of fexinidazole as observed in the first and second screenings. In this model, animals (10 per group) were infected with the VL-10 strain, and fexinidazole was administered on day 120 post-infection for 20 consecutive days. According to Coura et al. [15], two to three months after Chagas infection, the acute phase of the disease is followed by a long chronic period that is initially asymptomatic. This concept was validated by a group of experts during the Applied Research Meeting on Chagas disease, held in Araxá (Minas Gerais), Brazil [16]. Based on this understanding, we chose to start the chronic-phase treatment 120 days after infection. Before treatment, parasitic infection was confirmed by fresh blood examination (FBE) during the acute phase and by detection of anti-*T. cruzi* IgG antibodies in blood. Mice that were uninfected and infected but untreated acted as controls.

**Figure 2:**
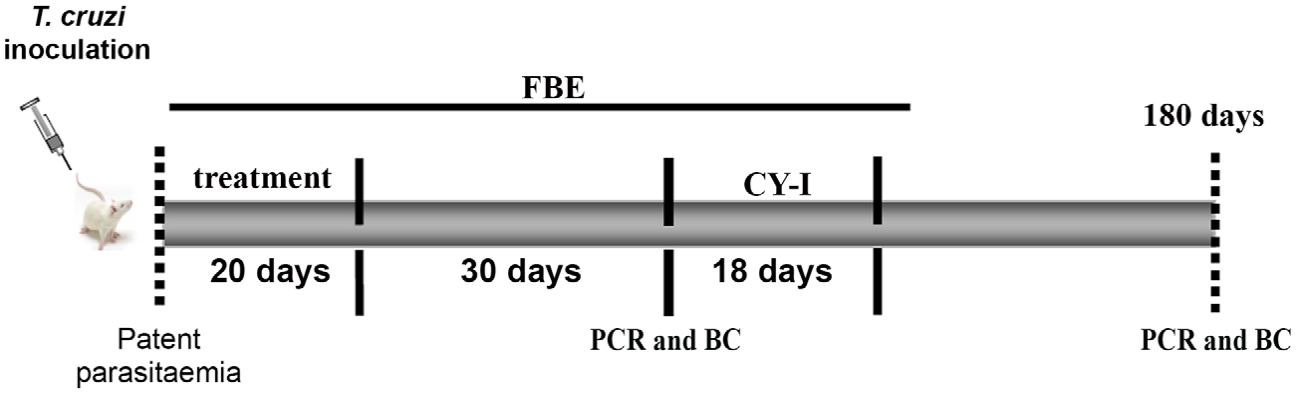
Timeline of chemotherapy experiments. Animals were inoculated with 5000 trypomastigotes of Y, CL, VL-10, or Colombian *Trypanosoma cruzi* strains, and treatment was started on the first day of detected parasitemia. During all treatments and up to 30 days post-treatment, parasitemia was evaluated by fresh blood examination (FBE) to determine the potential for natural reactivation of infection. Animals that did not present reactivation of parasitemia after treatment were submitted to immunosuppression consisting of three cycles of 50 mg of cyclophosphamide/kg (CY-I) of body weight for four consecutive days with intervals of three days between each cycle. Parasitemia was evaluated during the CY-I, as well as for the following 10 days after immunosuppression. Blood culture and PCR assays were performed 30 and 180 days post-treatment in mice with negative results in FBE before and after CY-I.

Parasitological cure was determined following the methodology standardized by Caldas et al. [17] based on a battery of three independent tests: FBE before and after cyclophosphamide immunosuppression (CyI), blood culture, and PCR assays performed on blood samples from mice with negative parasitemia. Animals showing negative results in the three tests were considered as cured (Figure 2).

For blood culture, animals that remained negative by FBE 30 and 180 days after treatment were bled from the orbital venous sinus and 400 µL of blood added in tubes containing 3 mL of LIT medium. The tubes were incubated at 28°C for 120 days and examined monthly for parasite detection.

For PCR assay, following the same procedure as for blood culture, 200 µL of collected blood was used for DNA extraction. DNA extraction and PCR were performed according to Gomes et al. [18] with some modifications. The primers used for the parasite minicircle amplification were: S35 5′- AAATAATGTACGGG(T/G)GAGATGCATGA-3′ and S36 5′-GGGTTCGATTGGGGTTGGTGT-3′ [19]. Thirty-five cycles of amplifications were carried out in a Research Programmable Thermal Controller (MiniCycler). The cycles consisted of a hold of 5 min at 95°C followed by 35 cycles of 1 min at 95°C for denaturation, 1 min at 65°C for primer annealing, and 1 min at 72°C for primer extension. Five microliters of the PCR product were analyzed by electrophoresis on a 6% polyacrylamide gel and visualized by silver staining.

Positive and negative blood samples and reagent controls were processed in parallel in each assay, and all experiments were conducted under controlled conditions. To avoid contamination, DNA extraction, mixing, and electrophoresis were performed in separate, delineated areas. To confirm the absence of inhibition factors, an internal control corresponding to a segment of the murine TNF-α gene was amplified [20].

### IgG Levels

Blood from treated mice was collected from the orbital venous sinus (500 µL) at 180 days after treatment. *T. cruzi* specific antibodies were detected by the technique described by Voller et al. [21]. Enzyme-linked immunosorbent assay plates were coated with *T. cruzi* antigen prepared from alkaline extraction from Y strain at exponential growth in LIT medium. Anti-mouse IgG-peroxidase conjugated antibody (Sigma Chemical Co.) was used. The mean absorbance for 10 negative control samples plus two standard deviations were used as the cut-off to discriminate positive and negative results.

### Myocardial Tissue Assessment

For histopathological analysis of myocardial tissues, mice were grouped into four categories – infected-treated-cured, infected-treated-not cured, infected-untreated (control), and uninfected (control) – and analyzed according to *T. cruzi* strain. Animals were sacrificed 180 days after treatment and heart tissues fixed with 10% formalin and embedded in paraffin. Blocks were cut into 4-µm sections and stained by hematoxylin–eosin (H&E) for inflammation assessment. Twenty fields from H&E slides were randomly chosen at 40× magnification for a total of 1.49×10^6^ µm^2^ analyzed myocardium area. Images were captured using a Leica DM 5000 B microcamera (Leica Application Suite, model 2.4.0R1) and processed with Leica Qwin V3 image analyzer software. The inflammatory process was evaluated by the correlation index among the number of cells observed in myocardium muscle from infected-treated-cured, infected-treated-not cured, infected-untreated, and uninfected animals [22].

### Ethics

All procedures and experimental protocols were conducted in accordance with COBEA (Brazilian School of Animal Experimentation) guidelines for the use of animals in research and approved by the Ethics Committee in Animal Research at UFOP (Protocol number 2009/17).

### Statistical Analysis

Histological and serological data were analyzed by nonparametric Tukey's Multiple Comparison Test. Difference was considered significant if the *P* value was less than or equal to 0.05.

## Results

**Figure 3:**
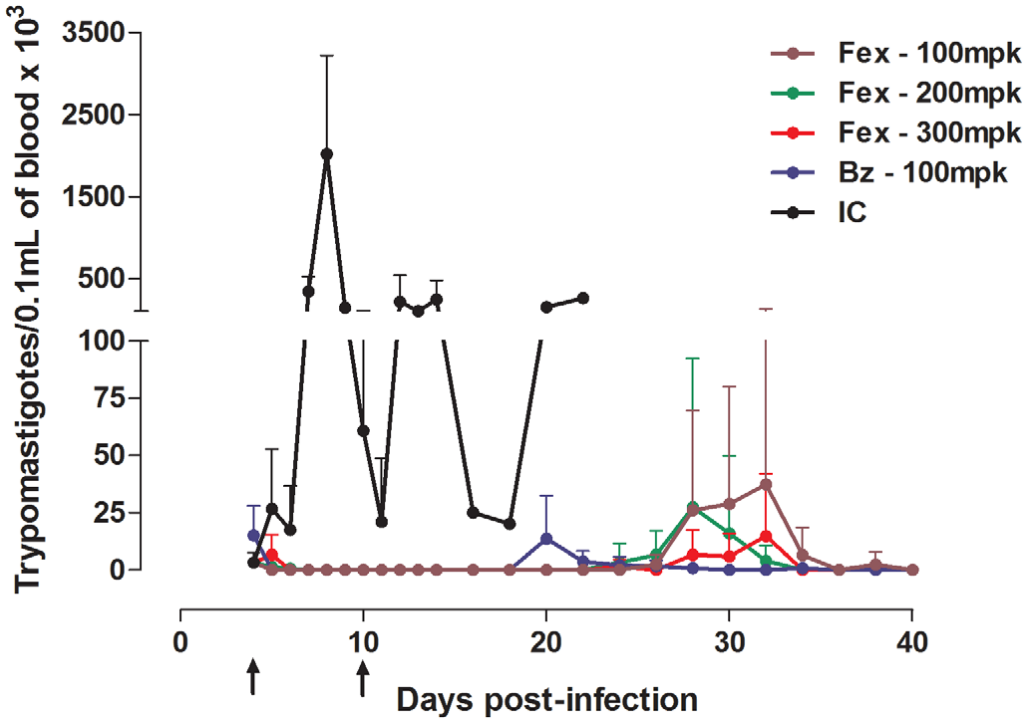
Parasitemia levels after 7 days of oral administration of fexinidazole. Parasitemia curve obtained from 6 mice infected with 5000 trypomastigotes of *T. cruzi* Y strain and treated daily with doses of fexinidazole 100, 200, and 300 mg/kg of body weight (mpk) or benznidazole 100 mpk for 7 consecutive days. Arrows indicate the first and the last day of treatment. IC: infected and untreated control.

To assess which dose-range of fexinidazole is able to suppress parasitaemia, animals infected with Y strain were treated with 100, 200, and 300 mpk of fexinidazole per day for 7 days. All doses tested resulted in a rapid suppression of the parasitaemia, similar to the standard benznidazole (Table 1, Figure 3). The 7-days treatment time however did not completely eliminate the parasites, and recrudescence of the parasitaemia occurred in all animals, yet slower in fexinidazole treated animals compared to benznidazole. While untreated animal all succumbed to the infection, all treatments were effective in preventing death (one animal died in the lowest fexinidazole dose).

**Table 1:** Biological parameters evaluated in mice infected with Y *Trypanosoma cruzi* strain and treated daily with 100, 200 or 300 mg/kg of body weight (mpk) of fexinidazole and 100 mpk of benznidazole for 7 days.

	Evaluated parameters		
Groups/n = 6	Clearance of parasitaemia mice/total (mean of doses±SD)	Time of parasitaemia clearance (mean of time in days±SD)	Mortality %*
Fexinidazole, 100 mpk	6/6 (1.0±0)	22.85±8.86	1/6 (16%)
Fexinidazole, 200 mpk	6/6 (1.66±0.82)	20.66±10.25	0/6 (0%)
Fexinidazole, 300 mpk	6/6 (1.5±0.55)	28.6±6.74	0/6 (0%)
Benznidazole, 100 mpk	6/6 (1.66±0.82)	11.5±9.16	0/6 (0%)
Control	-	-	6/6 (100%)

Treatments were started at 4^th^ day after infection.

* Until 30 days after treatment.

Based on these results, we evaluated the capacity of fexinidazole to induce parasitological cure in animals infected with Y strain over longer-term treatment. Infected animals were treated daily for 20 consecutive days using 50, 100, 200, and 300 mpk of fexinidazole, compared with 100 mpk of benznidazole. The time required to achieve parasite suppression by fexinidazole was dose-dependent, with the highest dose comparable to the standard benznidazole treatment (Table 2). When looking at the definitive clearance of parasitaemia through the combination of different methods, 80% of mice treated with fexinidazole 300 mpk were cured at the end of a 6-months follow up period versus 50% for benznidazole 100 mpk, fexinidazole 100 mpk and 200 mpk. These data suggest that a higher cure rate can be achieved in animals infected with *T. cruzi* Y strain with fexinidazole treatment, be it at a higher dose, than with the standard benznidazole dose of 100 mpk. All treatment regimens were effective in preventing death.

**Table 2:** Curative control of mice infected with Y strain of *Trypanosoma cruzi* and treated with fexinidazole 50, 100, 200, and 300 mg/kg of body weight (mpk) or benznidazole 100 mpk, for 20 consecutive days.

	Y strain - Treated groups				
Cure control	Fexinidazole				Benznidazole
(current methodology)	50 mpk/n = 10	100 mpk/n = 9	200 mpk/n = 9	300 mpk/n = 10	100 mpk/n = 20
Days of treatment for parasitemia negativation	3.77±1.82	2.44±1.13	1.89±0.6	1.2±0.42	1.19±0.68
Parasitemia reactivation after treatment	8/10	3/9	4/9	0/10	9/20
+Blood culture or PCR	0/2	1/6	1/5	2/10	1/11
Total of positive mice (%)	8/10 (80%)	4/9 (44%)	5/9 (55%)	2/10 (20%)	10/20 (50%)
Mortality*	0/10	0/9	0/9	0/10	0/20

+Blood culture or PCR = positive result for blood culture (HC) and/or blood PCR assay at 1^<$>\vskip 2\kern 1\raster(35%)="rg1"<$>st^ and 6^<$>\vskip 2\kern 1\raster(35%)="rg1"<$>th^ month post-treatment.

* Until 30 days after treatment.

We also assessed whether the highest dose of fexinidazole would also be more effective than standard benznidazole in treating mice infected with the benznidazole-susceptible CL strain, and the benznidazole-resistant VL-10 and Colombian strains of *T. cruzi* (Table 3). For the CL-strain, both the time to achieve parasitaemia suppression and the final cure rate were similar. While benznidazole could not suppress parasitaemia fully for the VL-10 strain, and cured none of the VL-10-infected animals, fexinidazole induced a rapid suppression of parasitaemia (Figure 3A), and cured 88.9% of the mice (Table 3). And while both benznidazole and fexinidazole rapidly suppressed parasitaemia in all animals infected with the Colombian strain, only fexinidazole could clear the parasitaemia resulting in a cure rate of 78% (Table 3). In the benznidazole-treated group, parasitaemia reappeared after the end of treatment starting on day 8 and persisted in all animals up to day 30 post-treatment (data not shown).

**Table 3:** Curative control of mice infected with CL, VL-10, and Colombian strains of *Trypanosoma cruzi* and treated during the acute phase of the infection with fexinidazole 300 mg/kg of body weight (mpk) and benznidazole 100 mpk for 20 consecutive days.

	Cure control				
*T. cruzi* strain: treatment	Parasitemia clearance (days of treatment)	+FBE	+ BC or PCR	+ Mice/total %	Mortality*
CL: fexinidazole, 300 mpk	1.5±0.53	0/10	2/10	2/10 (20%)	0/10
CL: benznidazole, 100 mpk	1.0±0	2/10	1/8	3/10 (30%)	0/10
VL-10: fexinidazole, 300 mpk	1.67±1.0	0/9	1/9	1/9 (11.1%)	0/9
VL-10: benznidazole, 100 mpk	ND	10/10	ND	10/10(100%)	1/10
Colombian: fexinidazole, 300 mpk	1.6±0.52	2/9	1/9	2/9 (22.2%)	0/9
Colombian: benznidazole, 100 mpk	2.1±1.66	10/10	ND	10/10(100%)	0/10

+FBE = fresh blood examination, positive parasitemia result.

+BC or PCR = positive result for blood culture (HC) and/or blood PCR assay at 1^st^ and 6^th^ month post-treatment.

* Until 30 days after treatment.

ND = not detected.

**Figure 4:**
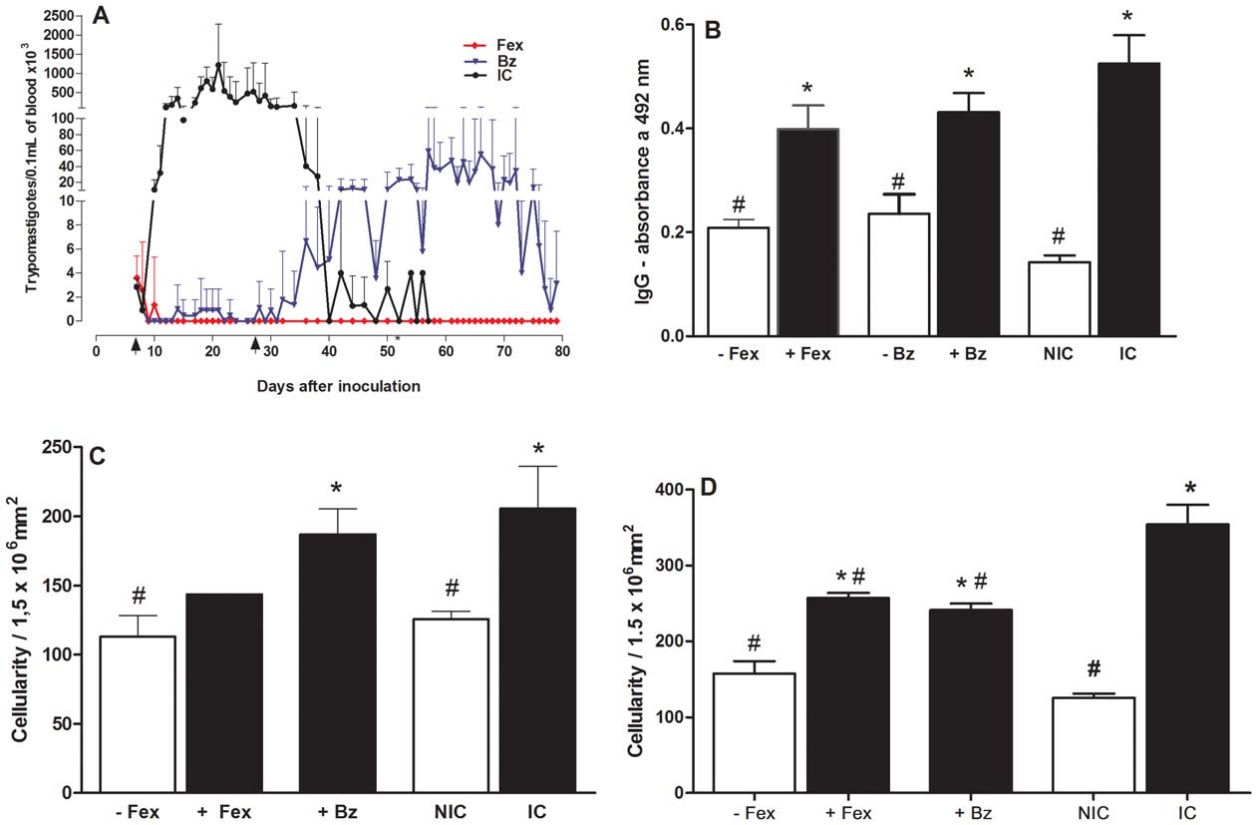
Anti-*Trypanosoma cruzi* activity of fexinidazole in mice infected with different parasite strains. Mice were inoculated with 5000 trypomastigotes of *T. cruzi* CL, VL-10, or Colombian strains by intraperitoneal route and treated with fexinidazole 300 mg/kg of body weight (mpk) or benznidazole 100 mpk for 20 consecutive days. A. Parasitemia curve from mice infected with VL-10 *T. cruzi* strain. Arrows indicate first and last days of treatment. B. IgG antibodies in mice inoculated with CL, VL-10, or Colombian *T. cruzi* strains, 6 months post-treatment. C. Myocardial inflammatory cell count in mice infected with VL-10 *T. cruzi* strain, 6 months post-treatment. D. Myocardial inflammatory cell count in mice infected with Colombian *T. cruzi* strain, 6 months post-treatment. −Fex = mice with parasite negativity in fresh blood examination, blood culture, and PCR assay; +Fex or +Bz = mice positive of parasites in fresh blood examination, blood culture, and PCR assay: NIC = non-infected control; IC = infected and untreated control. * Significant difference compared to NIC; # significant difference compared to IC.

Comparative analysis of specific *T. cruzi* antibodies detected in blood samples collected from mice inoculated with CL, VL-10, or Colombian *T. cruzi* strains, after 6 months post-treatment were performed. For these analyses, animals were classified based on results of parasitological and PCR evaluations: +fexinidazole or +benznidazole (mice treated with fexinidazole or benznidazole with positive parasitological and PCR results [parasitaemia present]); −fexinidazole or −benznidazole (mice treated with fexinidazole or benznidazole with negative parasitological and PCR results [no parasitaemia]); IC (infected and untreated controls); NIC (uninfected control mice). The −fexinidazole and −benznidazole animals had IgG antibody levels significantly lower than that of IC animals, and similar to healthy mice. In contrast, +fexinidazole or +benznidazole animals had antibody levels similar to that of IC mice (Figure 4B).

In order to evaluate the efficacy of early fexinidazole treatment in preventing the development of subsequent chronic myocardial lesions in mice infected with VL-10 and Colombian *T. cruzi* strain, quantitative analysis of inflammation of heart tissue in fexinidazole and benznidazole-treated mice was performed at 6 months post-treatment, and compared to heart tissue from untreated infected mice and health mice.

In mice infected with VL-10 strain, early fexinidazole treatment was shown to prevent or lessen the typical inflammation of heart tissue associated with the chronic phase of experimental Chagas disease, as illustrated by similar levels of inflammatory cells observed in the heart tissue of fexinidazole-treated and healthy animals (Figure 4C). In contrast, untreated mice and benznidazole-treated mice presented a higher number of inflammatory cells in their heart tissue (Figure 4C).

In mice infected with Colombian strain, treatment with either fexinidazole or benznidazole was shown to reduce the number of inflammatory cells in chronic phase of experimental Chagas disease, as illustrated by the significantly higher number of inflammatory cells in the hearts of untreated infected animals compared with fexinidazole- or benznidazole-treated animals (Figure 4D). Additionally, parasite detection was associated with inflammation intensity. Animals negative for *T. cruzi* (−fexinidazole) showed lower inflammation intensity in heart tissue than those positive of *T. cruzi* in parasitological or/and molecular tests. Also, no differences were observed in number of inflammatory cells between healthy and −fexinidazole treated mice (Figure 4D).

Based on the findings from treatment during acute-phase experimental Chagas disease, animals infected with VL-10 were treated during the chronic phase (120 days after inoculation) using the same treatment scheme. CyI treatment induced parasitemia reactivation in 20% (2/10) of animals treated with fexinidazole or benznidazole, and 60% (6/10) of untreated infected mice (Table 4). However, considering the results of blood culture and PCR assays performed at 30 and 180 days after treatment, therapeutic failure was detected in 30% (3/10) and 70% (7/10) of mice treated with fexinidazole and benznidazole, respectively (Table 4). The parasite or its kDNA was detected in 90% (9/10) of IC mice (Table 4).

**Table 4:** Curative control of mice infected with VL-10 strain of *Trypanosoma cruzi* treated during chronic-phase Chagas with fexinidazole 300 mg/kg of body weight (mpk) and benznidazole 100 mpk for 20 consecutive days.

	VL-10 strain		
Cure control	Fexinidazole 300 mpk	Benznidazole 100 mpk	IC
Parasitemia reactivation after treatment	2/10	2/10	6/10
+Blood culture or PCR	1/8	5/8	3/4
Total positive mice (%)	3/10 (30%)	7/10 (70%)	9/10 (90%)
Mortality*	0/10	0/10	0/10

IC: infected, untreated control group.

+BC or PCR = positive result for blood culture (HC) and/or blood PCR assay at 1^st^ and 6^th^ month post-treatment.

* Until 30 days after treatment.

**Figure 5:**
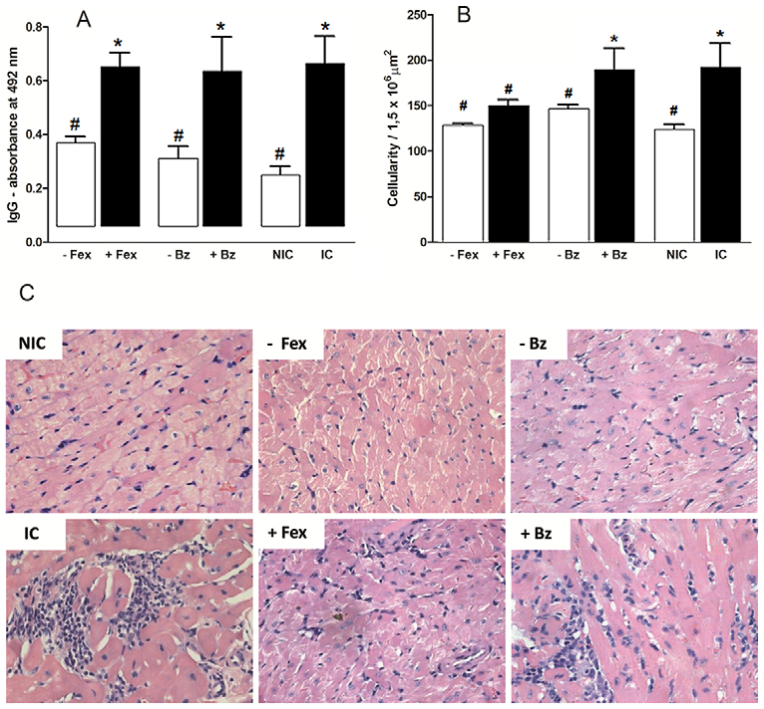
Effect of fexinidazole on chronic *Trypanosoma cruzi* infection. Mice were inoculated with 5000 trypomastigotes of VL-10 strain by intraperitoneal route and treated with fexinidazole 300 mg/kg of body weight (mpk), or benznidazole 100 mpk for 20 days. For controls, infected and untreated (IC) and uninfected (NIC) groups were also evaluated. A. IgG antibodies in mice treated with fexinidazole or benznidazole, compared with IC control group, 6 months post-treatment. B. Myocardial inflammatory cell count in cardiac tissue of mice infected with VL-10 *T. cruzi* strain, 6 months post-treatment. C. Hematoxilin-eosin stained slides. −Fex = mice with parasite negativity in fresh blood examination, blood culture, and PCR assay; +Fex or +Bz = mice positive of parasites in fresh blood examination, blood culture, and PCR assay: NIC = non-infected control; IC = infected and untreated control. * Significant difference compared to NIC; # significant difference compared to IC.

Six months after treatment of the chronic infection, animals that had been successfully treated with either benznidazole or fexinidazole (i.e. no more parasites detected) had reduced levels of specific anti-*T.cruzi* IgG antibody levels, comparable to healthy animals. In contrast, the antibody levels remained high and comparable to untreated controls in animals that were treated with either benznidazole or fexinidazole, but without clearing the parasitaemia (Figure 5A). Interestingly, fexinidazole treatment performed during the chronic phase of the infection significantly reduced the cardiac inflammation (Figures 5B and C), both in cured animals and in animals that hadn't fully cleared their parasitemia. In contrast, among the benznidazole-treated animals, a reduction of cardiac inflammation was detected only in those mice that cleared the parasitemia. Benznidazole-treated animals that did not clear their infection had similar levels of inflammation as untreated animals (Figure 5B and C).

This study confirms that fexinidazole is effective in curing experimental Chagas infection models, both in the acute and chronic stage, and including infections with benznidazole-resistant *T.cruzi* strains. At higher but well-tolerated doses, fexinidazole treatment achieves better cure rates and prevention of cardiac inflammation than the current standard treatment of benznidazole.

## Discussion

Current specific chemotherapy options for Chagas disease – benznidazole and nifurtimox – have limitations such as lack of effectiveness in achieving parasitological cure or in preventing the chronic phase of the disease. Fexinidazole, 5-nitroimidazole compound, was originally selected as a broad-spectrum antiprotozoal agent and has recently been rediscovered as a potential drug candidate for treatment of human African trypanosomiasis [11], [12], [23].

Previous studies had shown that fexinidazole has curative activity superior to benznidazole and nifurtimox during acute *T. cruzi* infection with the Brazil 32 strain in a murine model [12]. However, the methodologies used in this 1983 study for detecting parasites in the blood, including subinoculation in healthy mice, are no longer considered adequate to establish cure. We therefore re-evaluated the efficacy of fexinidazole in experimental Chagas disease using more sensitive state of the art methodologies (including blood culture, PCR and immunosuppression to reactivate lingering parasites), as well as using a range of *T. cruzi* strains with variable levels of resistance to benznidazole.

The rapid *in vivo* activity of fexinidazole conferring complete protection against death to infected mice when it is used in a short-term (7 days) treatment is in line with the activity originally reported by Raether et al. [12]. In our study, we additionally demonstrate that the anti-*T. cruzi* activity of fexinidazole was dose-dependent, and that a 20 days treatment with 100 or 200 mpk fexinidazole generated 50% definitive cure in mice infected with the benznidazole-susceptible Y strain, comparable to treatment with the standard dose of 100 mpk benznidazole. Moreover, a cure rate of 80% could be achieved when using 300 mpk of fexinidazole. Additionally, fexinidazole (300 mpk) and benznidazole (100 mpk) have similar activity against the susceptible benznidazole *T. cruzi* CL strain, but fexinidazole was highly effective in curing mice infected with the benznidazole-resistant VL-10 and Colombian strains.

Early fexinidazole treatment effectively prevented or reduced the development of myocarditis associated with chronic Chagas disease in mice infected with the benznidazole-resistant VL-10 and Colombian *T. cruzi* strains. Notably, when treated during the chronic phase of a *T.cruzi* VL-10 infection, fexinidazole but not benznidazole was also able to reduce myocarditis in all treated animals. This remarkable *in vivo* anti-*T. cruzi* activity of fexinidazole may be result of a combination of potent antiparasitic activity and advantageous pharmacokinetics properties. According Torreele et al. [11], fexinidazole is well absorbed after oral administration and broadly distributed to all organs and tissues in animals. This has implications for *in vivo* efficacy, considering that after parasitemia control, parasites may slowly replicate in various tissues.

Differing patterns of heart lesions were detected in benznidazole-treated animals inoculated with VL-10 and Colombian *T. cruzi* strains. A reduction in myocarditis in Colombian infected animals was observed, but this was not seen in those infected with VL-10 strain. During benznidazole treatment, animals infected with Colombian strain had negative fresh blood examination (data not shown), while those infected with VL-10 strain had positive parasitemia results. These results suggest that parasite load plays an important role in the pathogenesis of the chronic chagasic cardiomyopathy. This idea is corroborated by observations of Bustamante et al. [24] that re-exposure to the parasite through repeated infections aggravates heart dysfunction. Additionally, Caldas et al. [25] suggested that the efficacy of treatment in preventing chronic cardiac lesions is *T. cruzi* strain-dependent and probably related to the level of drug resistance to the parasite stock.

Specific anti-*T.cruzi* IgG levels detected 6 months after fexinidazole treatment were similar in non-infected control animals and definitively cured animals, and significantly lower than in non-treated infected mice or treated mice that remained parasitemic. The correlation between parasite detection and increased IgG levels is corroborated by a study by Garcia et al. [26], which demonstrated that in sera of benznidazole-treated mice, lower levels of antibodies specific to *T. cruzi* antigens are observed. According to the authors, reduction of antibody levels is possibly correlated with reduction of tissue damage, since antibodies against *T. cruzi* antigen may exert effects on cross-reactive epitopes on cardiac receptors, modulating the intensity of the heart tissue lesions. This study confirms a strong correlation between specific anti-*T.cruzi* IgG levels and intensity or cardiac inflammation, along with the capacity of fexinidazole treatment to reduce both.

It is important to note that fexinidazole was well tolerated by the *T. cruzi*-infected animals, and no adverse events were observed during the treatment period. These results are in agreement with others [11], [12] who demonstrated that fexinidazole was generally well tolerated after a single oral dose or repeated dosing, even at relatively high doses. Our safety findings agree with previous toxicologic studies that showed no effects on blood pressure, heart rate, and electrocardiogram intervals in dogs after single oral dose up to 1000 mg/kg. At the same dose in rats, no effects were observed on behavior characteristics or on respiratory parameters [11]. The No Observed Adverse Event Level (NOAEL) in 4-weeks repeated dose toxicokinetics studies in dogs and rats was set at 200 mg/kg/day [11].

In our study, fexinidazole blood levels were not measured, but others have shown that fexinidazole acts as a prodrug, which is oxidized *in vivo* to the more therapeutically relevant sulfoxide and sulfone metabolites [11], [27]. The sulfoxide metabolite reaches considerably higher levels in mouse blood than the parent compound, up to 40,000 ng/mL at 2 hours following oral dosing. Fexinidazole sulfone, the final metabolite to appear in the blood, continues to accumulate over 8 hours, reaching a concentration of 55,000 ng/mL. In mice, rats, and dogs, the half-life of fexinidazole after oral treatment ranges from 1 to 3 hours, while the half-life of the sulfoxide ranges from 2 to 7 hours and sulfone up to 24 h after dosing [11].

As earlier studies had shown mutagenic activity in the Ames test [12], the genotoxic profile of fexinidazole and its active metabolites was recently re-assessed in detail [28]. Fexinidazole is mutagenic in the classic Ames test; however, mutagenicity is either attenuated or lost in Ames Salmonella strains that lack one or more nitroreductase(s) [28]. It is known that these enzymes can nitroreduce compounds with low redox potentials such as fexinidazole, whereas their mammalian cell counterparts cannot. In order to specifically detect mammalian genetic toxicity, fexinidazole was tested in a panel of complementary assays, including an *in vitro* micronucleus test in human lymphocytes, an *in vivo* bone marrow micronucleus test in mice and an *ex vivo* unscheduled DNA synthesis in rats, and all were negative. Thus, it can be concluded that fexinidazole does not pose a genotoxic hazard to patients [10], [28].

Taken together, these results show that treatment with 300 mpk fexinidazole was able to produce high levels of parasitological cure in mice infected with benznidazole-susceptible, partially resistant, and resistant *T. cruzi* strains in acute and chronic phases of the experimental Chagas disease, and this is an improvement compared to the current standard treatment with benznidazole. This potent intrinsic and broad anti-*T. cruzi* activity, taken together with the fact that it is already in clinical development for another parasitic disease, suggests that fexinidazole is a promising drug candidate for human Chagas disease chemotherapy. Safety profile will need to be confirmed on further preclinical testing adapted to the longer treatment duration and Chagas disease presentation.
